# X‑Site Dependency of Optical and Electronic Properties in Ti_**3**_(C_2**–** ***y***_N_***y***_)T_***x***_ Carbonitride MXenes

**DOI:** 10.1021/acs.chemmater.5c02830

**Published:** 2026-01-07

**Authors:** Arunoda Lakmal, Augustus Figenshu, Sylvie Rangan, Christopher E. Shuck

**Affiliations:** † Department of Chemistry and Chemical Biology, 242612 Rutgers University, 123 Bevier Road, Piscataway, New Jersey 08854, United States; ‡ Department of Physics and Astronomy and Laboratory for Surface Modification, Rutgers University, 136 Frelinghuysen Road, Piscataway, New Jersey 08854, United States

## Abstract

MXenes, a family of two-dimensional transition metal carbides, nitrides, and carbonitrides with the general formula of M_*n*+1_X_*n*_T_*x*_ (where M represents an early transition metal, X is C and/or N, and T_*x*_ is the surface functional groups), offer exceptional tailorability in structure, composition, and surface chemistry. Among them, nitrogen-containing MXenes have enhanced electronic and optical properties compared to their carbon analogs. Yet, challenges in synthesizing them have made nitride and carbonitride MXenes the least explored class. Herein, we report the synthesis of Ti_3_Al­(C_2–*y*_N_*y*_) MAX phases using a high-aluminum method to minimize oxygen impurities as well as other competing binary and ternary phases. Therein, subsequent etching and delamination of Ti_3_Al­(C_2–*y*_N_*y*_) MAX phases into Ti_3_(C_2–*y*_N_*y*_)­T_*x*_ MXenes were done using a coupled HF/HCl/LiCl method. Systematic variation of X-site chemistry (Ti_3_C_2_T_*x*_, Ti_3_C_1.75_N_0.25_T_*x*_, Ti_3_C_1.5_N_0.5_T_*x*_, Ti_3_C_1.25_N_0.75_T_*x,*_ and Ti_3_CNT_*x*_) enabled direct correlations between chemistry and optoelectronic properties. Increased nitrogen content leads to increased preference for halogen terminations, stronger light-matter interaction, blue-shifted optical absorbance, and decreased electrical conductivity. Despite these variations, the work function remains nearly constant across all compositions, indicating that it is primarily dictated by M and T_*x*_ chemistries. These findings demonstrate that solid-solution carbonitride MXenes provide a platform to independently control optical and electronic behaviors, offering opportunities for MXene-based optoelectronic and energy applications.

## Graphical abstract

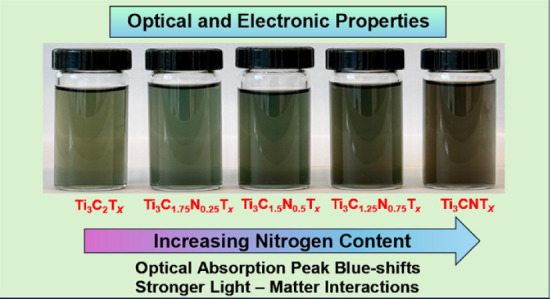

## Introduction

Two-dimensional (2D) materials such as graphene and its derivatives, hexagonal boron nitride (h-BN), and transition metal dichalcogenides (TMDs) have garnered significant attention from the scientific community.^1^ Among all 2D materials discovered to date, MXenes are the fastest-growing class due to their structural and chemical diversity.^2,3^ MXenes include 2D carbides, nitrides, and carbonitrides with the general formula of M_*n*+1_X_*n*_T_*x*_ (*n* = 1–4) where M, X, and T_*x*_ represent an early transition metal (Ti, V, Nb, Mo, etc.), C and/or N, and the surface termination groups (typically O, OH, F, Cl), respectively.^4^ Since the initial report of the first MXene in 2011, research has primarily focused on Ti_3_C_2_T_*x*_, including synthesis, properties, and applications, demonstrating tremendous promise in a multitude of applications.^5–7^ Broadly, MXenes have been used in fields such as electrochemical energy storage,^8^ electromagnetic interference (EMI) shielding,^9^ functional textiles,^10^ optoelectronics,^11^ etc.

A significant benefit of MXenes is the tailorability of their chemistry at either the M, X, or T_*x*_ site. Significant progress has been made in understanding the effect of M-site chemistry on the resultant MXene properties.^12–15^ By adjusting M-site chemistry, a variety of structures have been discovered, including solid-solution,^16,17^ ordered-double transition metal,^18,19^ in-plane ordered structures,^20,21^ and even high-entropy MXenes.^22^ However, the effect of X-site substitution in MXenes remains less understood. One year after the initial discovery of MXenes, Ti_3_CNT_*x*_ was synthesized from its parent MAX precursor, Ti_3_AlCN.^23^ Since then, many research groups have synthesized nitrogen-containing MAX phases such as V_2_GaN, V_2_Ga­(C_1–*y*_N_*y*_), V_2_GeN, V_2_Ge­(C_0.5_N_0.5_), Ti_2_Al­(C_1–*y*_N_*y*_), Ti_3_Al­(C_2–*y*_N_*y*_), Ti_4_Al­(C_3–*y*_N_*y*_), and others.^24–27^ Compared to pure carbide MAX phases, nitrogen-containing MAX phases are more difficult to synthesize. Carbide MAX phases are thermodynamically more stable due to stronger carbide bonds and are favored during high-temperature synthesis. Furthermore, nitride MAX phases compete with stable binary metal nitrides; therefore, reactions often lead to the formation of binary phases instead of ternary MAX phases.^24,25^ This results in limiting the structural diversity of pure nitride and carbonitride MAX phases. With this limited structural diversity, only a few successful demonstrations of etching and delamination have been reported.^28,29^ Moreover, reported properties vary, potentially due to enhanced hydrolysis in solution^30^ or increased oxygen in the X-sublattice.^31^

Despite this, carbonitrides have already demonstrated unique functionalities: Ti_3_CNT_*x*_ was found to have a more negative zeta potential compared to Ti_3_C_2_T_*x*_,^32^ and was shown to have anomalously high absorption of electromagnetic waves.^9^ Jindata et al. reported the shift in Ti 3p core levels of Ti_3_CNT_*x*_ to a lower binding energy compared to Ti_3_C_2_T_*x*_, which is due to negative electronic compressibility (NEC).^33^ Density functional theory (DFT) calculations attribute this to the nature of Ti–N bond hybridization, which enhances the ability of Ti orbitals to accommodate additional electrons above the Fermi level, thereby producing the NEC effect, offering unique opportunities in energy and charge-storing applications.^33,34^ Ti_2_C_0.5_N_0.5_T_*x*_ was shown to have higher capacitance than Ti_2_CT_*x*_ for Na-ion batteries.^35^ It has been suggested that Ti_3_CNT_*x*_ will exhibit higher electrical conductivity relative to Ti_3_C_2_T_*x*_ due to higher free electron density. However, the electrical conductivity of Ti_3_CNT_*x*_ was found to be an order of magnitude lower than Ti_3_C_2_T_*x*_.^28^ Furthermore, Ti_3_CNT_*x*_ has shown higher optical absorption in the near-infrared (NIR) region of the electromagnetic spectrum, which makes it an attractive material for photothermal therapy.^28,36^

Recently, a range of promising carbonitride MAX phases, Ti_2_Al­(C_1–*y*_N_*y*_), Ti_3_Al­(C_2–*y*_N_*y*_), and Ti_4_Al­(C_3–*y*_N_*y*_), were reported, and their analogous multilayered MXenes were synthesized using a molten salt etching method.^26^ Ti_3_(C_2–*y*_N_*y*_)­T_*x*_ compounds are a unique model system, within which the controlled carbon substitution with nitrogen into X-sites should enable tailoring their resultant electronic and optoelectronic properties. Therefore, in this work, single flakes of Ti_3_(C_2–*y*_N_*y*_)­T_*x*_ are synthesized using a scalable aqueous etching with hydrofluoric (HF) and hydrochloric (HCl) acid, alongside simultaneous delamination using lithium chloride (LiCl). A systematic X-site compositional change is achieved in Ti_3_(C_2–*y*_N_*y*_)­T_*x*_ solid-solution MXenes, including Ti_3_C_2_T_*x*_, Ti_3_C_1.75_N_0.25_T_*x*_, Ti_3_C_1.5_N_0.5_T_*x*_, Ti_3_C_1.25_N_0.75_T_*x,*_ and Ti_3_CNT_*x,*_ allowing for direct study of the impact of the X-site on bulk and surface properties of the resultant MXenes. We find that increasing nitrogen content leads to a higher preference for halogen surface terminations, enhanced light-matter interaction with a monotonic blue-shift in optical absorbance, and reduced electrical conductivity. Despite these systematic optoelectronic variations, we find that there is little variation in work function (both in UHV and air) across all chemistries, indicating that M-site and surface terminations are the primary determinants of the work function. This study establishes X-site engineering as a viable strategy to decouple and independently control optical and electronic properties, offering new opportunities to rationally design MXenes with targeted functionalities.

## Experimental Section

### Synthesis of Ti_3_Al­(C_2–*y*_N_*y*_) MAX Phases

A series of different MAX phases was synthesized to include *y* = 0, 0.25, 0.5, 0.75, and 1. All MXenes were synthesized using a top-down approach, where the corresponding MAX phases, Ti_3_Al­(C_2–*y*_N_*y*_), were synthesized using a solid phase reaction at an elevated temperature inside a tube furnace. Metal powder precursors: Ti (Thermo Scientific, −325 mesh, 99.5%), Al (Thermo Scientific, −325 mesh, 99.5%), Aluminum Nitride (Atlantic Equipment Engineers, −325 mesh, 99.9%) and Graphite (Thermo Scientific, 7–11 μm, 99%) were mixed in M/A/X molar ratio of 3.25:2.2:2 and ball milled using Alumina milling balls at 200 rpm for 24 h followed by passivation (allowing the precursor powder to settle with slow exposure to air). Powders (10.0 g each) were then loaded into Alumina crucibles and annealed in a high-temperature tube furnace (TF 1700, Across International) at 3 °C/min to 1380 °C and held for 3 h. Then the furnace was heated at a rate of 5 °C/min to 1480 °C and held for 1 h before cooling to room temperature. Both heating, dwelling, and cooling steps were done under an ultrahigh-purity argon (Airgas) atmosphere to prevent oxidation. Then the sintered block was crushed with a mortar and pestle, and the synthesized MAX phase powder was stirred with a 5-fold diluted (a volume of 80 mL deionized water:20 mL concentrated HCl) HCl acid (Thermo Scientific, 37 vol %) solution overnight to dissolve any intermetallic impurities. The resulting powder was washed and neutralized with deionized water with the aid of vacuum-assisted filtration (Millipore polycarbonate, 0.2 μm pore size). The solid residue containing the purified MAX phase was oven-dried at 60 °C overnight. Dried MAX phase powder was sieved, and particles sized 20–38 μm were used for etching.

### Synthesis of MXenes

One gram of as-synthesized MAX phase was added to a 2:2:6 volumetric mixture of hydrofluoric (HF) acid (Thermo Scientific, 48–51%): deionized water: hydrochloric (HCl) acid (Thermo Scientific, 37%) for a total volume of 20 mL. To the same reaction, 2.10 g of LiCl dissolved in DI water (4 mL) was added slowly. The reaction mixture was stirred at 500 rpm at 45 °C for 24 h. At the end of stirring, the solutions were centrifuged for 15 min at 8000 rpm, and the supernatant was discarded until it became dark in color. Once the supernatant became dark, successive centrifugation cycles were repeated at 3500 rpm for 10 min to collect the delaminated MXene flakes.

### Synthesis of Free-Standing MXene Films

The delaminated MXene flakes were further concentrated by centrifugation at 12000 rpm for 1 h. The sediment was redispersed in DI water to achieve a final concentration between 2 and 4 mg mL^–1^. A volume of 5–10 mL of solution (depending on the starting concentration) was filtered through a polycarbonate filter paper (Isopore, 47 mm, 0.2 μm pore size) and vacuum-dried overnight.

### Thin Film Fabrication

Cover glasses (Corning, 25 × 25 mm) were cleaned by bath sonication (Symphony, VWR) in Alkonox detergent for 15 min, followed by sequential rinsing in a DI water bath, ethanol bath, again in a DI water bath, and air-dried. To make the surface more hydrophilic before depositing MXene solutions, the glass slides were exposed to mixed O_2_/Ar plasma treatment at 200 W for 5 min (PE 25, Plasma Etch). The thin films were fabricated by spin coating 1–2 mL of the concentrated MXene solutions at 8000 rpm for 5-minute intervals.

### Characterization of MAX Phases and MXenes

X-ray diffraction (XRD) measurements were conducted on MAX phase powder samples and MXene free-standing films using a Bruker D2 Phaser diffractometer operating at 30 kV and 10 mA with a Cu Kα source. Free-standing MXene films were vacuum-dried overnight before performing XRD measurements. Data was collected from 4 to 80° (2θ) with a step size of 0.02° and a rate of 0.5 s per step. Slight texturing was applied to all MAX phase samples to confirm that only Ti_3_Al­(C_2–*y*_N_*y*_) phases are present. The *a* and *c-*lattice parameters and overall crystal structure were evaluated using Rietveld refinement with GSAS-II software.^37^

X-ray photoemission spectroscopy (XPS) was performed using a Thermo K-Alpha instrument equipped with a monochromatized Al Kα X-ray source (hν = 1486.7 eV) and with an energy resolution of 0.6 eV for the core levels. All powder samples and films were mounted on conductive carbon tape. Core levels analysis was performed using the Avantage 6.7.0 software, with a fitting scheme similar to that of a recent review.^38^ The use of a flood gun was necessary for some powder MAX phases. For consistency, binding energies were rigidly shifted so as to place the Ti–C component of the C 1s region at 282.0 eV, in accordance with previously reported results.^38^ Herein is reported the elemental and chemical analysis of both MAX and MXene phases.

### Optical Properties

UV–vis-NIR spectra of delaminated colloidal Ti_3_(C_2–*y*_N_*y*_)­T_*x*_ MXene solutions were recorded using a spectrophotometer (Agilent Cary 60) in the 200–1000 nm range. The concentration of the colloidal MXene solutions was calculated by filtering a known volume of the concentrated solution through a polycarbonate filter paper (Isopore, 47 mm, 0.2 μm pore size), which was then vacuum-dried. The mass of the free-standing films was measured, and the concentration was recorded in units of mg mL^–1^. The concentration of the colloidal solutions ranged from 2 to 4 mg mL^–1^. Necessary dilutions were done before UV–vis measurements, where the final concentrations of the diluted solutions used for spectroscopy measurements were around 0.01–0.04 mg mL^–1^.

Absorption and reflectance measurements of MXene thin films in the UV–vis-NIR spectral range were carried out using an integrating sphere attached to a spectrophotometer (UV 2600i-Plus, Shimadzu) in the 200–1400 nm range. Necessary baseline corrections were performed before each measurement.

### Electrical Conductivity Measurements

The electrical conductivity of the free-standing MXene films was measured using a four-point probe (Jandel RM3000+). Measurements were made at 10 mA on both forward and reverse bias. Sheet resistance: R_s_ (Ω/□) at a minimum of 10 different locations of the film was read using the instrument, and the thickness of the MXene films was measured at a minimum of 10 different locations using a high-accuracy digital micrometer (Mitutoyo d_2_ with minimum thickness measurement of 0.1 μm).

### Work Function and Surface Potential Measurements

Work function measurements were carried out on the conductive MXene films in a Thermo ESCALAB 250xi instrument, housing both X-ray and UV-photoemission spectroscopies (XPS and UPS), with 0.6 and 0.1 eV resolution, respectively. For consistency, core levels and valence band were measured in XPS (*hν* = 1486.7 eV), followed by UPS valence band (*hν* = 40.8 eV) and work function measurements (*hν* = 21.1 eV). A reference metallic Fermi edge was also measured on the sputtered-clean stainless-steel sample holder in electrical contact with the MXene films. All films were found metallic with a well-defined Fermi edge and did not necessitate charge compensation. Binding energies are referenced to the Fermi level set at 0 eV. Secondary electron cutoffs (SECO) were determined using a linear extrapolation of the secondary electron edge to the background, measured on biased samples (−10 V). The work function is obtained as WF = hν – (SECO – Fermi Level) with hν = 21.2 eV.

Kelvin probe force microscopy was carried out with the Electrostatic Force Microscopy (EFM) mode using an Asylum Research Cypher ES atomic force microscope using overall platinum-coated electrically conductive cantilevers, 240 AC-PP (MikroMasch). The measurements were carried out under ambient conditions in the sealed atomic force microscope enclosure to reduce noise at a set sample temperature of 25 °C. The spin-coated MXene thin films were grounded using conductive tape to the AFM’s ground. A digital resolution of 512 × 512 points with a scan area of 5 μm × 5 μm and a scanning rate of 0.75 Hz was used. The oscillation frequency of the probe was set at or near resonance at 70 kHz. The AFM images were processed using the Gwyddion software.

## Results and Discussion

### MAX Phases

Successful synthesis of Ti_3_Al­(C_2–*y*_N_*y*_) MAX phases was confirmed by X-ray diffraction (XRD) (Figure 1a). In the carbonitride MAX phases, with increasing nitrogen content, the crystal structure must deform to accommodate the lower atomic radius of nitrogen compared to carbon. As reported by the literature,^26^ this was evidenced by the shift in the (110) peak (Figure 1b) to a higher 2θ angle. Slight texturing was applied to all MAX phases for XRD to confirm the presence of only one MAX phase.^39^ The absence of any additional peaks in the region of 5 to 15° 2θ confirms that the MAX phases contain only respective Ti_3_Al­(C_2–*y*_N_*y*_) phases (Figure S1). For the Ti_3_Al­(C_2–*y*_N_*y*_) system, the *a* and *c*-lattice parameters as well as the overall crystal structure were analyzed using the GSAS II Rietveld refinement software (Figure S2 and Table S1). The *a*-lattice parameters varied from 3.084 Å (Ti_3_AlC_2_) to 3.044 Å (Ti_3_AlCN), whereas the *c*-lattice parameters ranged from 18.63 Å (Ti_3_AlC_2_) to 18.40 Å (Ti_3_AlCN) as nitrogen content increased according to Vegard’s law (Figures 1c and d). A previous study demonstrated the effect of using excess aluminum during the synthesis of MAX phases on improved crystallinity and attaining an ideal 3:2 titanium: carbon stoichiometry.^40^ This was because excess molten Al enhanced the diffusion of reactants during the annealing process, as well as the excess Al reacting with oxygen to make Al_2_O_3_, which will be isolated from the MAX phases.^40^ Therefore, in the current study, an excess amount of Al was used in synthesizing all the MAX phases compared to conventional carbonitride synthesis, and unlike any previous studies, a quantitative trend in both *a* and *c-*lattice parameters was observed, likely due to the lack of oxygen in the sublattice.^26,31,40^ However, with the addition of nitrogen as X-site elements, traces of cubic Ti­(C_1–*y*_N_*y*_) appeared, which suggests that the binary cubic phases become increasingly thermodynamically favorable with increasing nitrogen (Figure S2).^26^ A few attempts were made to synthesize Ti_3_Al­(C_2–*y*_N_*y*_) carbonitride phases, where *y* > 1, and all transformed into either a mixture of Ti_3_Al­(C_2–*y*_N_*y*_) and Ti_4_Al­(C_3–*y*_N_*y*_) or purely Ti_4_Al­(C_3–*y*_N_*y*_) phases (Figure S3). Therefore, this study is continued with the Ti_3_Al­(C_2–*y*_N_*y*_) carbonitride system, where the y value ranges from 0 to 1.

**Figure 1:**
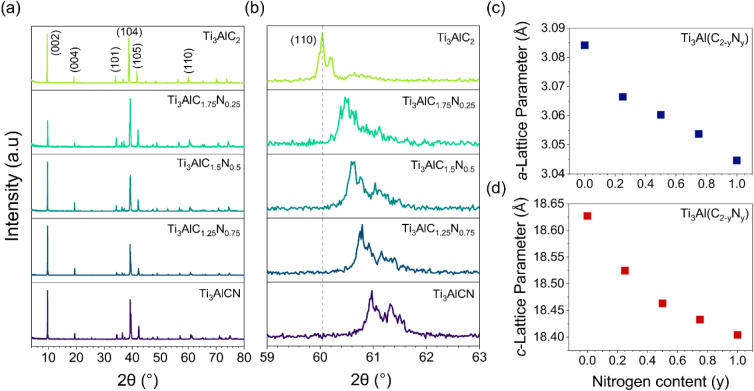
X-ray diffraction patterns (XRD) of Ti_3_Al­(C_2–*y*_N_*y*_) carbonitride MAX phases from y = 0 to 1 and respective *a* and *c*-lattice parameters. (a) Patterns from 5 to 80° showing the (00*l*), (10*l*), and (110) reflections. (b) Enlarged 2θ area from 59 to 63° showing the gradual shift of the (110) reflection with increasing N content. (c) *a*-Lattice parameters plotted as a function of N content. (d) *c*-Lattice parameters plotted as a function of N content.

Elemental quantification and chemical assessment of the MAX phases was performed using XPS. The C/N stoichiometry at the X-site of the MAX phase was quantified using the measured Ti 2p, Al 2p, C 1s, and N 1s core levels of Figure S6. As expected, examination of the carbon and nitrogen intensities across the MAX phases with changing compositions reveals a consistent trend. Experimentally determined compositions of carbonitride MAX phases display a slight overestimation of nitrogen (carbon deficiency) compared to the target compositions (Table S2).

### MXenes

The Ti_3_(C_2–*y*_N_*y*_)­T_*x*_ MXenes were synthesized by selective etching of Al layers from their respective MAX phase precursors using a mixture of hydrofluoric (HF) and hydrochloric (HCl) acid solutions, alongside simultaneous delamination using lithium chloride (LiCl). This combined approach led to an increased single flake yield of all carbonitrides. Thus, for consistency, MXenes from Ti_3_C_2_T_*x*_ was synthesized using the same HF/HCl/LiCl method. The increased yield and consistency may be due to enhanced etching on the MAX phase surface, facilitated by simultaneous delamination, or due to the dependency of delamination on pH. The first effect was observed in previous reports, indicating that the etching kinetics of Ti_3_AlC_2_ are reaction interface-limited.^41^ However, the effect of the pH on delamination needs to be studied further. The delaminated MXenes were fabricated into freestanding films with the aid of vacuum filtration. The purity of the MXene films was assessed by performing powder X-ray diffraction. All films exhibited (00*l*) reflections with no higher order peaks corresponding to unetched MAX and multilayered MXenes (Figure 2a). Variation of the (002) peak position could be attributed to the presence of different amounts of intercalated water molecules within flakes.^39^ Elemental composition as well as bonding environment were studied using XPS. The systematic variation of the C/N ratio, as well as the composition of the surface terminations, was determined from the Ti 2p, C 1s, N 1s, O 1s, F 1s, and Cl 2p core levels analysis of Figures S7 and S8. Examining the C 1s core levels, a systematic decrease in the intensity of the peak component at 282.0 eV, which corresponds to the titanium–carbon (Ti–C–Ti) bonding, was observed (Figure 2b). This suggests the effective decrease of carbon atoms in the X-site, varying from Ti_3_C_2_T_*x*_ to Ti_3_CNT_*x*_. A similar trend was observed for the N 1s region, where the intensity of the peak component at 397.0 eV, which corresponds to the titanium–nitrogen (Ti–N–Ti) bonding, increased from Ti_3_C_2_T_*x*_ to Ti_3_CNT_*x*_. There is also a slight increase in oxidation (TiO_2_) as the nitrogen content increases. The experimentally obtained C/N stoichiometry at the X-site aligns well with nominal values and is represented in Table S3. These results indicate that the observed oxidation does not affect the reported compositional trends, and that the use of these MXenes to study X-site-dependent optical and electronic properties is appropriate.

**Figure 2:**
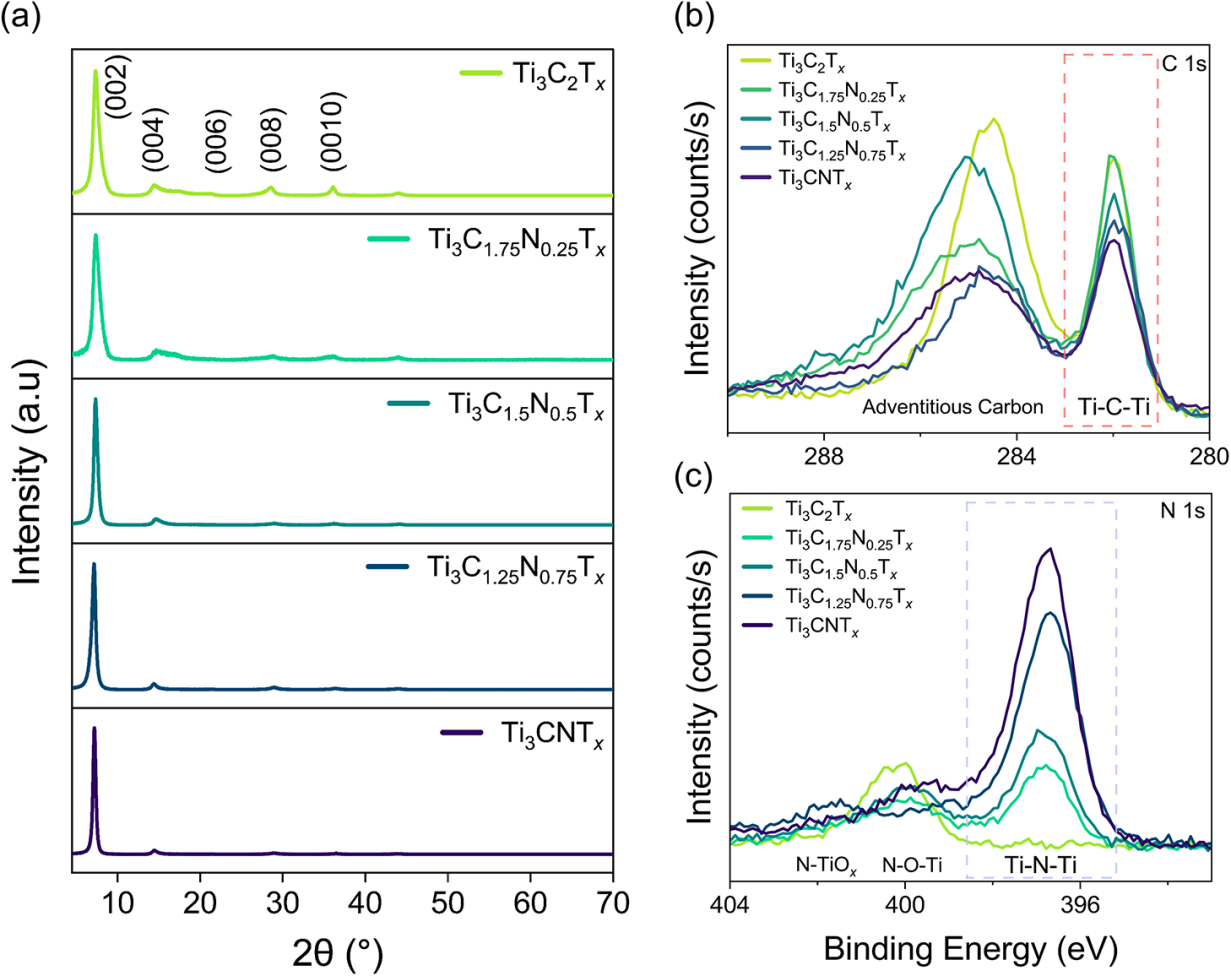
XRD of Ti_3_(C_2–*y*_N_*y*_)­T_*x*_ MXenes from y = 0 to 1 and respective C 1s and N 1s core level XPS spectra. (a) Patterns from 5 to 70° showing the (00*l*) reflections corresponding to highly in-plane oriented MXene flakes. The absence of any higher-order peaks indicates the proper delamination of the MXenes to single flakes. (b) C 1s and (c) N 1s core level XPS spectra plotted as a function of the N content demonstrate the reduction of the intensity of the Ti–C–Ti peak component (282.0 eV) as well as the increase in the intensity of the Ti–N–Ti peak component (397.0 eV) with increasing N ratios.

The quantification of surface terminations is vital for predicting properties of MXenes.^42^ The majority of the surface terminations were found to be oxygen-related and fluorine surface terminations, with a minority of chlorine (Figure 3). This aspect has been previously observed for MXenes etched in acidic solutions.^43^ In all Ti_3_(C_2–*y*_N_*y*_)­T_*x*_ structures, chlorine was present in minor quantities, and its relative amount gradually increased from 10.1% (Ti_3_C_2_T_*x*_) to 20.8% (Ti_3_CNT_*x*_). For fluorine terminations, relative amounts did not show a simple monotonic trend but showed increased fluorine contents in carbonitride MXenes compared to Ti_3_C_2_T_*x*_. The sum of O/OH terminations also showed a decrease in carbonitride MXenes compared to Ti_3_C_2_T_*x*_ (Table S4). This trend can be attributed to the increased electronegativity of nitrogen relative to carbon, which alters the local electronic environment of the transition metal sites.^32,44^ Nitrogen substitution makes the metal atoms more electropositive, thereby stabilizing the adsorption of halogen species, which show less directional bonding compared to strong directional M=O bonds.^43^ In this way, varying the C/N ratio tunes the surface termination balance by modulating the oxidation state and binding preference of surface Ti atoms.

**Figure 3:**
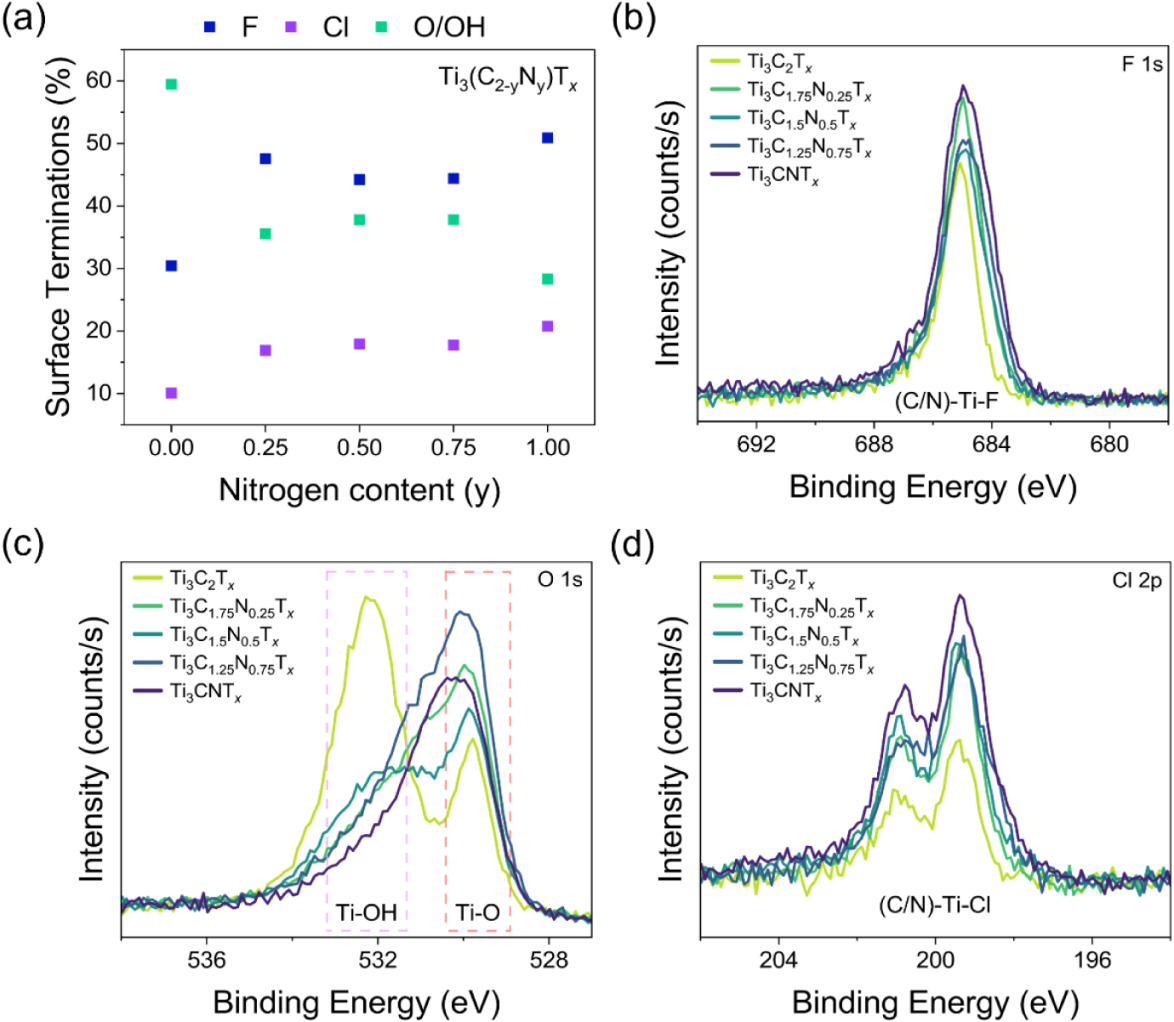
Quantification of the surface terminations in Ti_3_(C_2–*y*_N_*y*_)­T_*x*_ MXenes from y = 0 to 1 using core level XPS. (a) Relative quantities of fluorine, chlorine, and the sum of both oxygen and hydroxyl terminations are represented as a percentage where the *x* is assumed to be 2. XPS spectra plotted as a function of the N content. (b) F 1s, (c) O 1s, and (d) Cl 2p. The highlighted regions in the O 1s spectra correspond to the binding energies of Ti–O and Ti–OH surface termination components.^38^

The delaminated MXenes were readily dispersible in water and showed a gradual change in color from green (Ti_3_C_2_T_*x*_) to grayish brown (Ti_3_CNT_*x*_), as shown in Figure 4a. The gravimetric extinction coefficients (ε) measured over a wavelength spanning the ultraviolet–visible-near-infrared (UV–vis-NIR) spectrum are given in Figure 4b. The intensity of the extinction features in the UV region varied in the range of 4000–10000 mL mg^–1^ m^–1^ for Ti_3_(C_2–*y*_N_*y*_)­T_*x*_ MXenes. There is a direct monotonic trend where the maximum extinction coefficient increases with nitrogen content, suggesting that there are more allowed electronic state transitions available for carbonitride MXenes compared to Ti_3_C_2_T_*x*_. This stronger light-matter interaction also suggests that N-rich MXenes couple more efficiently with photons. In the visible to NIR region, compositional variation resulted in strong extinction peaks spanning the entire range, with extinction coefficients lower than the interband transitions. Ti_3_C_2_T_*x*_ exhibited an optical absorption peak at λ_max_ = 750 nm as reported previously in the literature.^45^ The respective peak shifted to lower wavelengths (blue-shift) with increasing nitrogen; Ti_3_C_1.75_N_0.25_T_*x*_ (λ_max_ = 700 nm), Ti_3_C_1.5_N_0.5_T_*x*_ (λ_max_ = 668 nm), and Ti_3_C_1.25_N_0.75_T_*x*_ (λ_max_ = 640 nm). Ti_3_CNT_*x*_ showed a broad diminished absorption peak in the visible spectrum region, confirmed by previous studies.^28^ A study conducted by Salles et al., where the visible absorption peak shifted reversibly from 770 to 670 nm, probing the electrochromic effect in acidic electrolytes, suggested the protonation and deprotonation of oxygen-containing surface termination groups.^46^ A similar assumption can be made with the observed shifts in the optical absorption peak, where there could be a systematic variation in the O/OH ratio. Another reason may be the influence of the electron-withdrawing effect of nitrogen atoms, modifying the oxidation states of the titanium atoms. Deconvolution of the O 1s signal into precise O/OH components is challenging due to method limitations and requires more advanced characterization.^38^ However, there is a tendency to alter the titanium oxidation states, thereby altering the surface electron density, which shifts the location of the plasmon resonance.^46^

**Figure 4:**
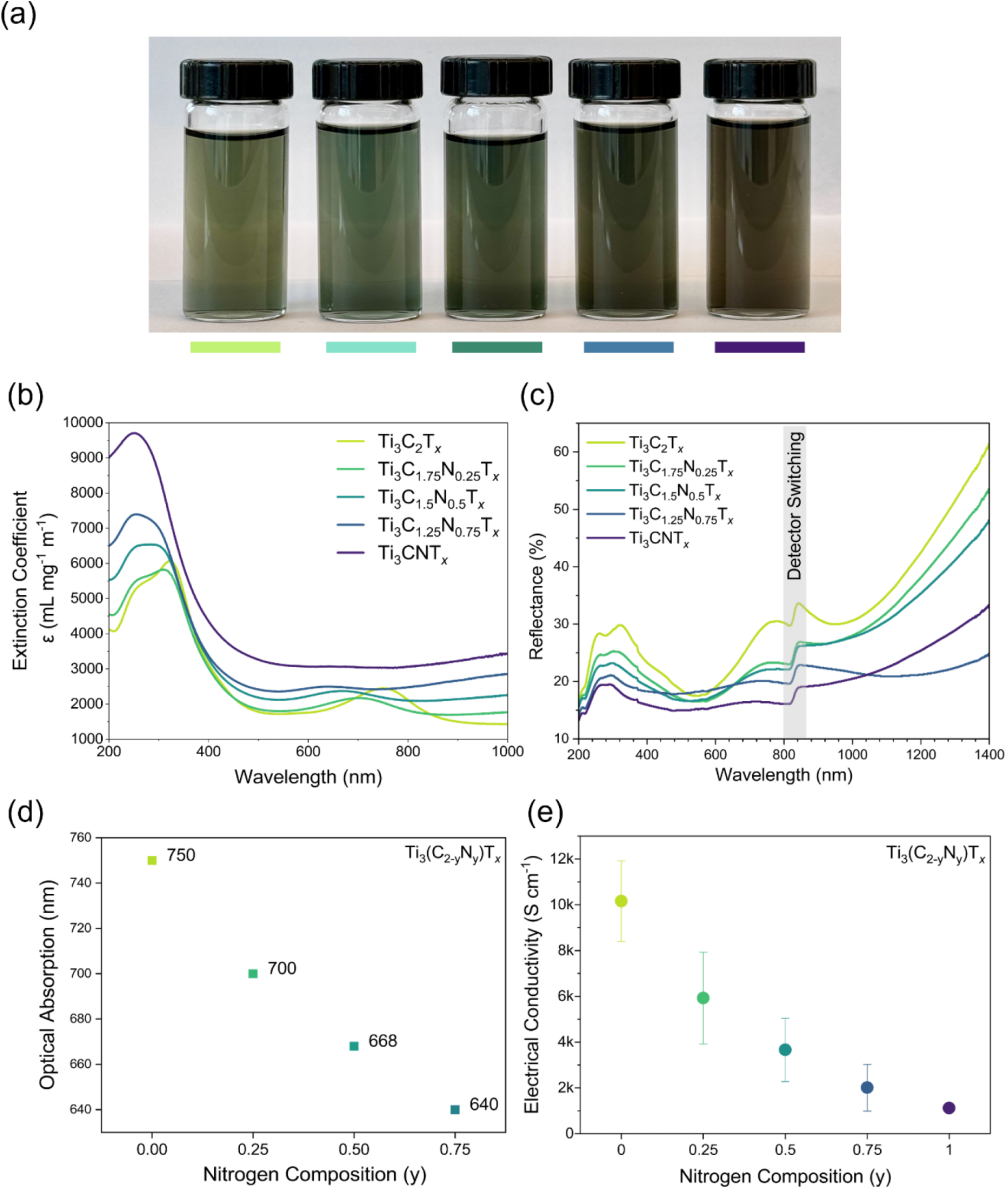
Optical properties and electrical conductivities of Ti_3_(C_2–*y*_N_*y*_)­T_*x*_ MXenes. (a) The colloidal solutions of MXenes in deionized water in the order of Ti_3_C_2_T_*x*_, Ti_3_C_1.75_N_0.25_T_*x*_, Ti_3_C_1.5_N_0.5_T_*x*_, Ti_3_C_1.25_N_0.75_T_*x*_, and Ti_3_CNT_*x*_ from left to right. A gradual change in color from green (Ti_3_C_2_T_*x*_), via emerald-green to grayish brown (Ti_3_CNT_*x*_), resulting from the variation of C/N ratio in the X-site, can be observed. (b) UV–vis-NIR optical absorption properties of Ti_3_(C_2–*y*_N_*y*_)­T_*x*_ colloidal MXenes. (c) Total reflectance of MXene thin films spin-coated on a glass substrate, over the UV–vis-NIR spectrum. This is the sum of specular reflectance and diffuse reflectance and was measured with respect to a BaSO_4_ pressed pellet. The artifact in the 820–840 nm region is due to the difference in detection, caused by the switching of the detector from a photomultiplier tube to a lead sulfide detector. (d) Optical absorption maxima (λ_max_) of Ti_3_(C_2–*y*_N_*y*_)­T_*x*_ MXenes with varying nitrogen compositions. All except Ti_3_CNT_*x*_ showed a prominent optical absorption peak in the visible spectrum region. (e) Electrical conductivity of Ti_3_(C_2–*y*_N_*y*_)­T_*x*_ MXene free-standing films with varying nitrogen compositions. The location of the data point represents the mean conductivities calculated from sheet resistance and film thickness. The error bars represent the respective errors associated with the conductivity calculations.

To investigate the optical properties of thin films of carbonitride MXenes, colloidal solutions were spin-coated on glass slides. The absorption properties over wavelength spanning the UV–vis-NIR spectrum showed a similar trend to that of colloidal solutions (Figure S9). Reflectance measurements were taken over a wavelength spanning the UV–vis-NIR spectrum with the aid of an integrating sphere (Figure 4c and Figure S10). The reflectance measurements in the UV region showed a clear trend where Ti_3_C_2_T_*x*_ MXene showed the highest reflectance, around 30% with respect to barium sulfate (BaSO_4_) pressed pellets. The reflectance of carbonitride MXenes decreased with increasing nitrogen ratios, which aligns well with the complementary absorption properties of carbonitride MXenes. In the spectral region of 500–900 nm, Ti_3_C_2_T_*x*_ shows a strong reflectance, which diminishes with increasing nitrogen content. Beyond 900 nm, all MXenes showed an exponential increase in reflectance within the region of investigation. However, the rate of increase was comparatively low for carbonitride MXenes compared to Ti_3_C_2_T_*x*_.

To assess the effect of nitrogen substitution on electrical conductivity, the sheet resistance of each film was measured using the four-point probe method. The variation of electrical conductivity with varying nitrogen ratios is given in Figure 4e. Ti_3_C_2_T_*x*_ showed a maximum electrical conductivity of 11700 S cm^–1,^ and the conductivities of carbonitrides were lower than Ti_3_C_2_T_*x*_. Ti_3_CNT_*x*_ showed a maximum conductivity of 1250 S cm^–1^, which is an order of magnitude less than pure carbide MXenes. A summary of the electrical conductivity measurements is given in Table S7. Furthermore, the physical appearance of free-standing Ti_3_(C_2–*y*_N_*y*_)­T_*x*_ MXene films fabricated by vacuum filtration showed a gradual shift in the reflectance color from dark purple (Ti_3_C_2_T_*x*_) to black (Ti_3_CNT_*x*_) (Figure S11). Also, the metallic luster of MXene films decreased from Ti_3_C_2_T_*x*_ to Ti_3_CNT_*x*_, which suggests a decrease in free carrier concentrations.

The work function of the Ti_3_(C_2–*y*_N_*y*_)­T_*x*_ MXene films was investigated using ultraviolet photoelectron spectroscopy (UPS). Figure 5a shows the SECO and VB spectra obtained on each film, referenced to the Fermi level (set at 0 eV of binding energy) of a sputter-cleaned metallic surface (stainless steel) in contact with the measured samples. A distinct Fermi edge in the valence band spectra is a clear indication of the metallic character of the synthesized MXenes (Figure 5a). Ti_3_C_2_T_*x*_ has a work function of 4.0 ± 0.1 eV, and all carbonitride MXenes showed slightly lower work functions compared to Ti_3_C_2_T_*x*_. Irrespective of the C/N ratio, work functions of 3.8 ± 0.1 eV were measured (Table 1). The nitrogen substitution is therefore not directly affecting the work function. In light of previous reports on MXenes, the work function seems to be more impacted by surface terminations. Schultz et al. investigated the work function of Ti_3_C_2_T_*x*_ as a function of annealing temperature.^47^ In their study, the room temperature work function was found to be 3.9 eV, matching our work function measurements. With annealing, the desorption of fluorine terminations increased the work function up to 4.1 eV. A similar kind of surface termination dependency was also experimentally proven by a different study, which achieved a work function variation exceeding 0.6 eV depending on different levels of doping at the surface.^48^ This means that one can alter the bulk properties of the MXene phases with N insertion for the desired composition and yet still be able to control the work function independently via surface terminations.

**Figure 5:**
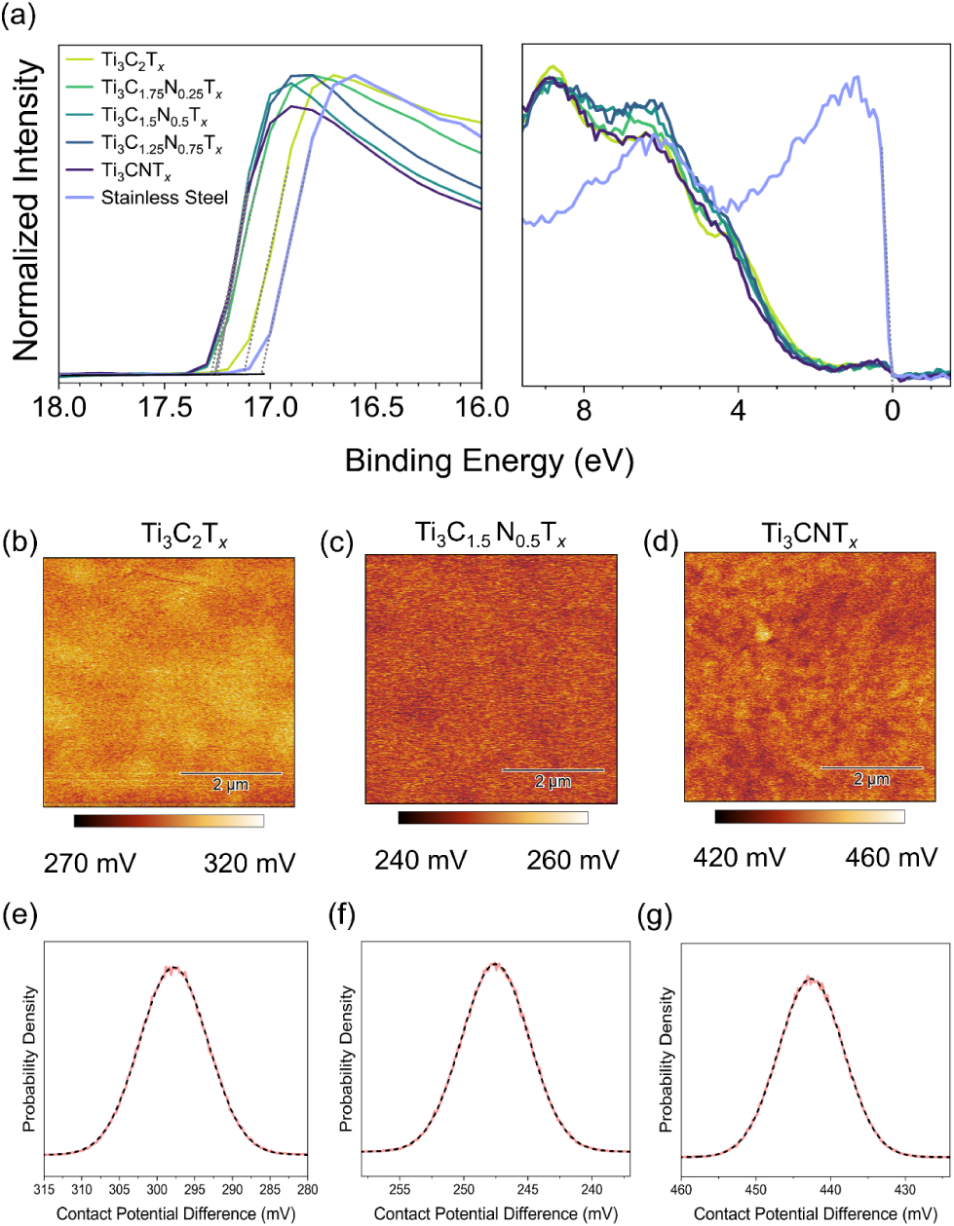
Work function and surface potential measurements of Ti_3_(C_2–*y*_N_*y*_)­T_*x*_ MXene films. (a) SECO and VB regions measured for each film (with the Fermi level set as 0 eV). (b), (c), and (d) show the spatially resolved surface potential distribution measured using Kelvin probe force microscopy in Ti_3_C_2_T_*x*_, Ti_3_C_1.5_N_0.5_T_*x*_, and Ti_3_CNT_*x*_, respectively. The scan area was 5 × 5 μm, and the scale bar represents the potential in mV. The corresponding distribution of surface potentials compared to a Gaussian distribution is shown in (e) Ti_3_C_2_T_*x*_, (f) Ti_3_C_1.5_N_0.5_T_*x*_, and (g) Ti_3_CNT_*x*_.

**Table 1:** MXene Work Functions Measured from UPS

Composition	Work Function (eV)
Ti_3_C_2_T_*x*_	4.0 ± 0.1
Ti_3_C_1.75_N_0.25_T_*x*_	3.8 ± 0.1
Ti_3_C_1.5_N_0.5_T_*x*_	3.8 ± 0.1
Ti_3_C_1.25_N_0.75_T_*x*_	3.8 ± 0.1
Ti_3_CNT_*x*_	3.8 ± 0.1

The spatial resolution of the surface potential was investigated using Kelvin probe force microscopy (Figure 5b–d). All MXenes showed a positive potential with respect to the AFM cantilever and a homogeneous distribution of surface potential. Quantitative surface potential did not show any linear trend with the C/N compositional variations, in agreement with the similar work functions measured in UPS at the MXenes surface. The Gaussian distribution of surface potential indicates uniform charge distribution, suggesting that both X-site composition and surface terminations are randomly distributed rather than spatially ordered (Figure 5e–g).^49^

## Conclusion

This study demonstrates the successful synthesis of Ti_3_(C_2–*y*_N_*y*_)­T_*x*_ MXenes, obtained from their respective parent Ti_3_Al­(C_2–*y*_N_*y*_) MAX phase precursors. MAX precursors were obtained using an improved Al-rich method allowing for a controlled X-site C/N ratio in a series of compounds. Additionally, simultaneous etching and delamination with HF/HCl/LiCl of the precursors led to an improved yield and consistency for carbonitride MXene synthesis. N-rich MXenes are found to have a higher preference for halogen surface terminations than pure carbide MXenes, underscoring the influence of X-site chemistry on resultant surface functionalization. Optical characterization revealed enhanced light-matter interaction and a systematic blueshift in the absorbance with increasing nitrogen content. Reflectance measurements on MXene films showed three prominent reflectance features in the UV–visible-NIR regions, and all exhibited higher reflectance toward the NIR region, with the magnitude decreasing with nitrogen content. The electrical conductivity decreased with increasing nitrogen, consistent with reduced carrier concentration or shorter carrier lifetimes. In contrast, UPS and KPFM measurements showed nearly constant work functions regardless of N content, suggesting that surface terminations, rather than X-site substitution, primarily dictate electronic levels.

Overall, this study demonstrates that X-site chemistry provides a powerful tool to modify the optical and electronic properties fundamentally different than surface terminations or M-site chemistry. Solid-solution carbonitrides, in particular, present a promising route to tailor light-matter interactions and bulk functional behavior for optoelectronic and energy applications.

## Supplementary Material



## Notes

Experimental procedures for the synthesis, etching, and delamination of Ti_3_(C_2–*y*_N_*y*_)­T_*x*_ carbonitride MXenes; powder X-ray diffraction (XRD) patterns and Rietveld refinements of the Ti_3_Al­(C_2–*y*_N_*y*_) MAX phase precursors; additional XRD analyses illustrating phase evolution as a function of nitrogen content and synthesis temperature; fitted X-ray photoelectron spectroscopy (XPS) core-level spectra and peak assignments for both MAX phases and MXenes; compositional analysis of C/N ratios and surface terminations derived from XPS; optical absorption of MXene thin films; schematics of the integrating sphere reflectance geometry; photographs of free-standing MXene films; atomic force microscopy (AFM) and Kelvin probe force microscopy (KPFM) measurements of surface topography and potential distributions; and statistical summaries of electrical conductivity measurements; tables summarizing crystallographic parameters, compositional analyses, surface termination fractions, XPS fitting parameters, and electrical transport data (PDF)
