# Supplementary material for: X‑Site Dependency of Optical and Electronic Properties in Ti 3 (C2– y N y )T x Carbonitride MXenes
Source: Chem Mater. 2026 Jan 7;38(2):890–9. doi: 10.1021/acs.chemmater.5c02830 (PMC12854704; doi:10.1021/acs.chemmater.5c02830)
Supplement: Supplementary file 1 [file cm5c02830_si_001.pdf]

Supporting Information

X-Site Dependency of Optical and Electronic Properties in $Ti_3(C_{2-y}N_y)T_x$ Carbonitride MXenes

Arunoda Lakmal[1], Augustus Figenshu[1], Sylvie Rangan[2], Christopher E. Shuck[1]*

[1]Department of Chemistry and Chemical Biology, Rutgers University, 123 Bevier Road, Piscataway, New Jersey 08854, United States

[2]Department of Physics and Astronomy and Laboratory for Surface Modification, Rutgers University, 136 Frelinghuysen Road, Piscataway, New Jersey 08854, United States

***Corresponding Author: Christopher.E.Shuck@rutgers.edu**

Etching and delamination of MXenes

1 g of each synthesized MAX phase was etched with a mixture of 2:2:6 volumetric ratio of hydrofluoric (HF) acid (Thermo Scientific, 48-51%): deionized water: hydrochloric (HCl) acid (Thermo Scientific, 37%) for a total volume of 20 mL to etch Al layers topochemically. All reactions were conducted at 40 °C for 24 h, constantly stirring at 500 rpm. This led to multilayered (ML)-$Ti_3(C_{2-y}N_y)T_{x,}$ and the etched solution was washed with deionized water several times with centrifugation at 5000 rpm for 10 min until neutral pH. A small amount of ML-$Ti_3(C_{2-y}N_y)T_x$ was collected by vacuum filtration to perform powder X-ray diffraction (PXRD), and the rest was used for delamination while still wet. To delaminate MXenes into single flakes, 2.10 g of Lithium Chloride (LiCl) was dissolved in 10 mL of DI water in a PTFE (Nalgene) bottle, and wet ML MXenes were transferred to it. The reaction mixture was stirred at 500 rpm at 40 °C for 24 h. At the end of stirring, the solutions were centrifuged for 15 minutes at 6000 rpm until the supernatant became dark in color. Once the supernatant became dark, successive centrifugation cycles were repeated at 3500 rpm for 10 minutes to collect the delaminated MXene flakes. This approach did not yield significant amounts of delaminated single flakes. Therefore, all MXenes were etched and delaminated using the procedure given in the experimental section.

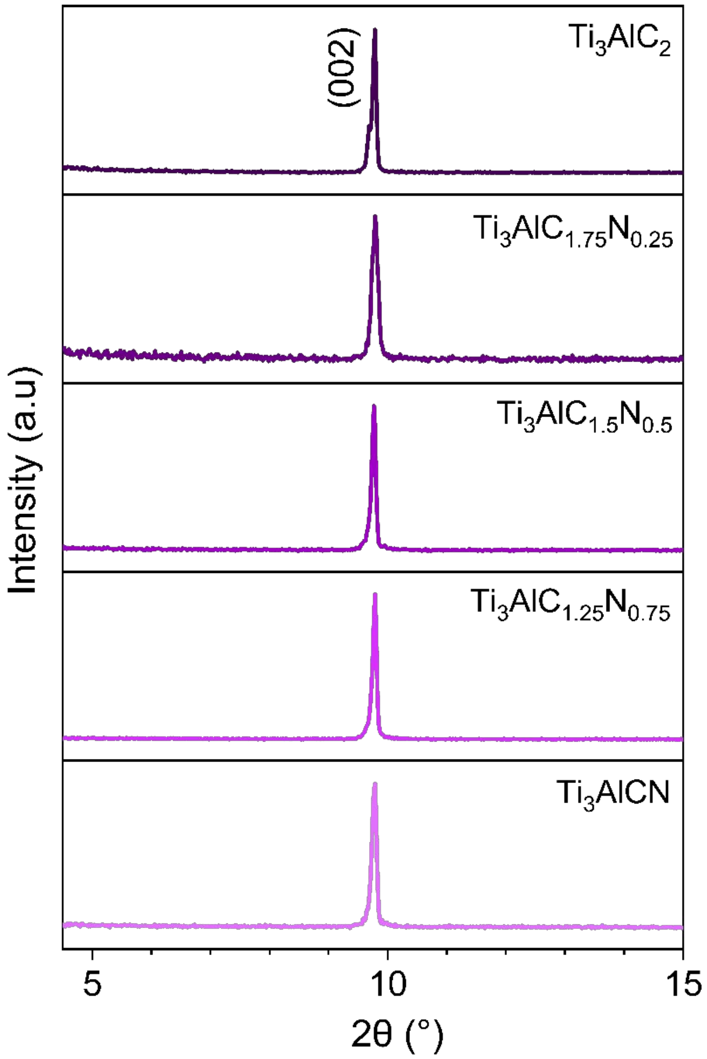

Figure S1. X-ray diffraction pattern of $Ti_3Al(C_{2-y}N_y)$ MAX phases from 4.5° to 15° 2θ. The presence of only one (002) peak around 9.8° suggests the existence of only $Ti_3Al(C_{2-y}N_y)$ phases.

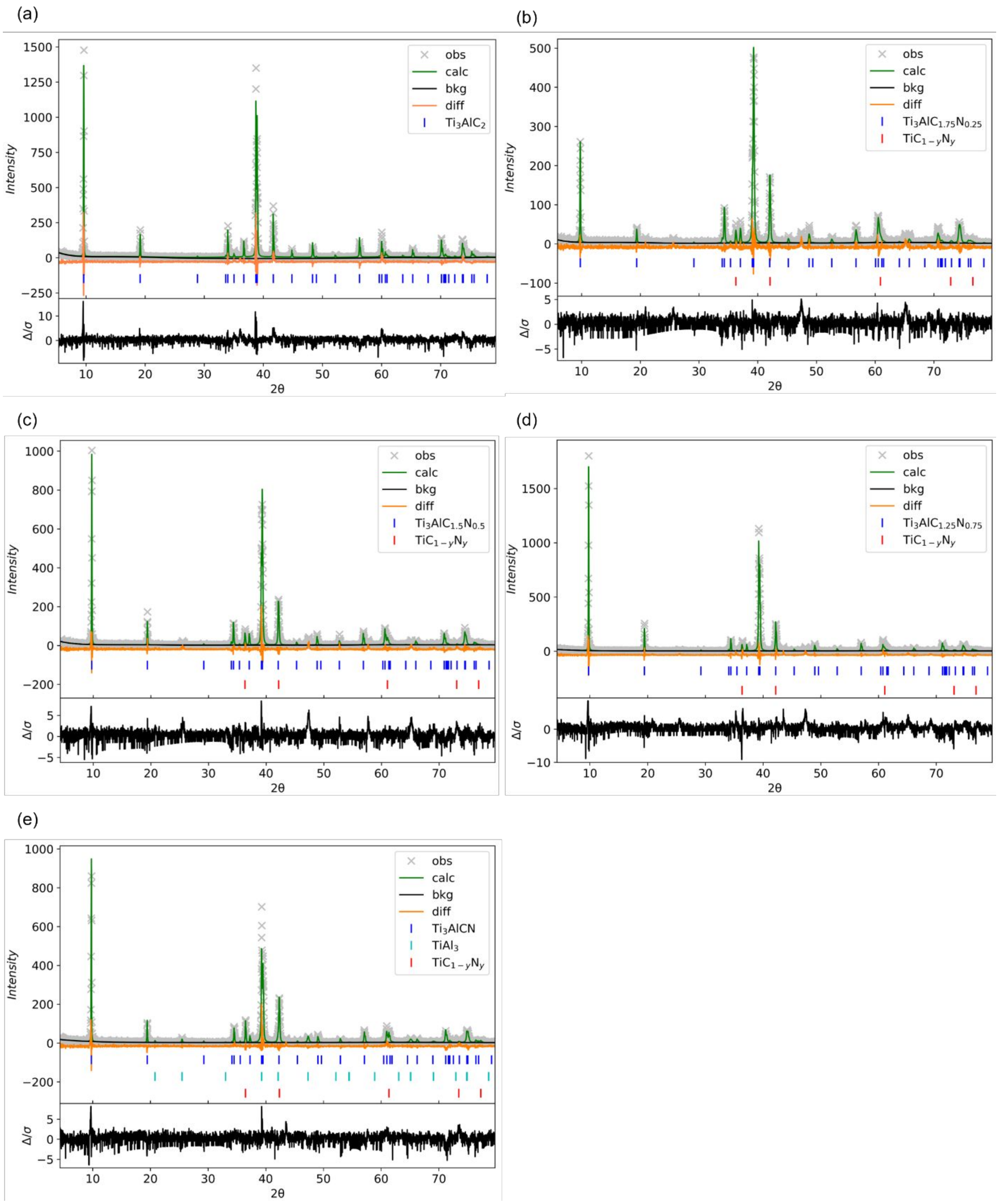

Figure S2. The Rietveld refinement of all the MAX phases in the carbonitride system was performed using GSAS II software.[1] Figures S2 (a-e) show the refinement results and point to the coexistence of different minority phases in (a) Ti_3AlC_2, (b) $Ti_3AlC_{1.75}N_{0.25}$, (c) $Ti_3AlC_{1.5}N_{0.5}$, (d) $Ti_3AlC_{1.25}N_{0.75,}$ and (e) Ti_3AlCN, respectively. The coexisting phases are indicated

underneath each MAX phase. Traces of cubic Ti($C_{1-y}N_y$) were observed for all nitrogen-substituted compounds.

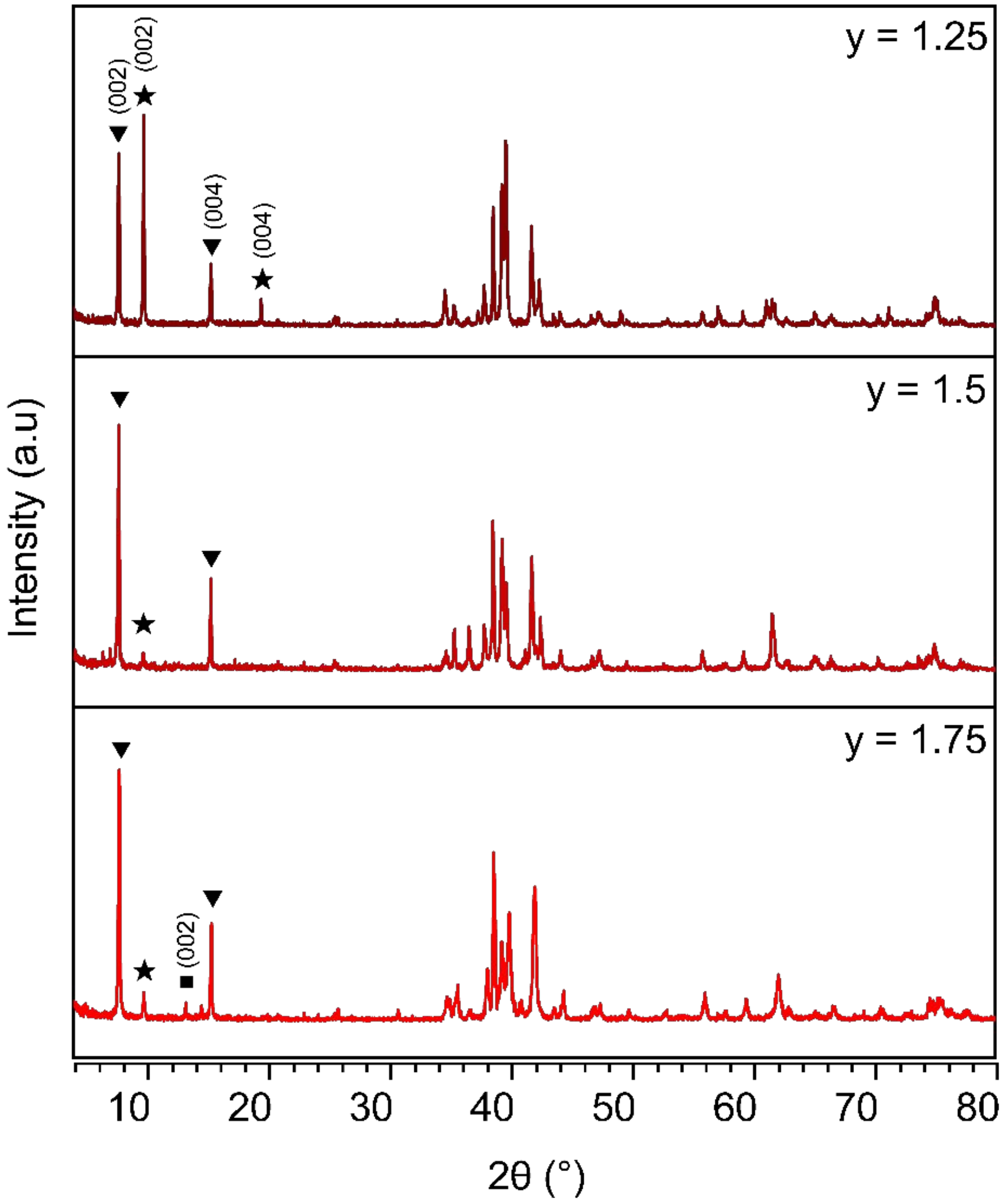

Figure S3. X-ray diffraction pattern of $Ti_3Al(C_{2-y}N_y)$ MAX phases for y > 1. The XRD pattern representing y = 1.25 contains both $Ti_4Al(C_{3-y}N_y)$ (black inverted triangle) and $Ti_3Al(C_{2-y}N_y)$ (black star) MAX phases. In y =1.5, the major phase is $Ti_4Al(C_{3-y}N_y)$ with lesser $Ti_3Al(C_{2-y}N_y)$ phase. In addition, y =1.75, shows a similar composition to the previous except it has a lesser amount of $Ti_2Al(C_{1-y}N_y)$ (black square).[2]

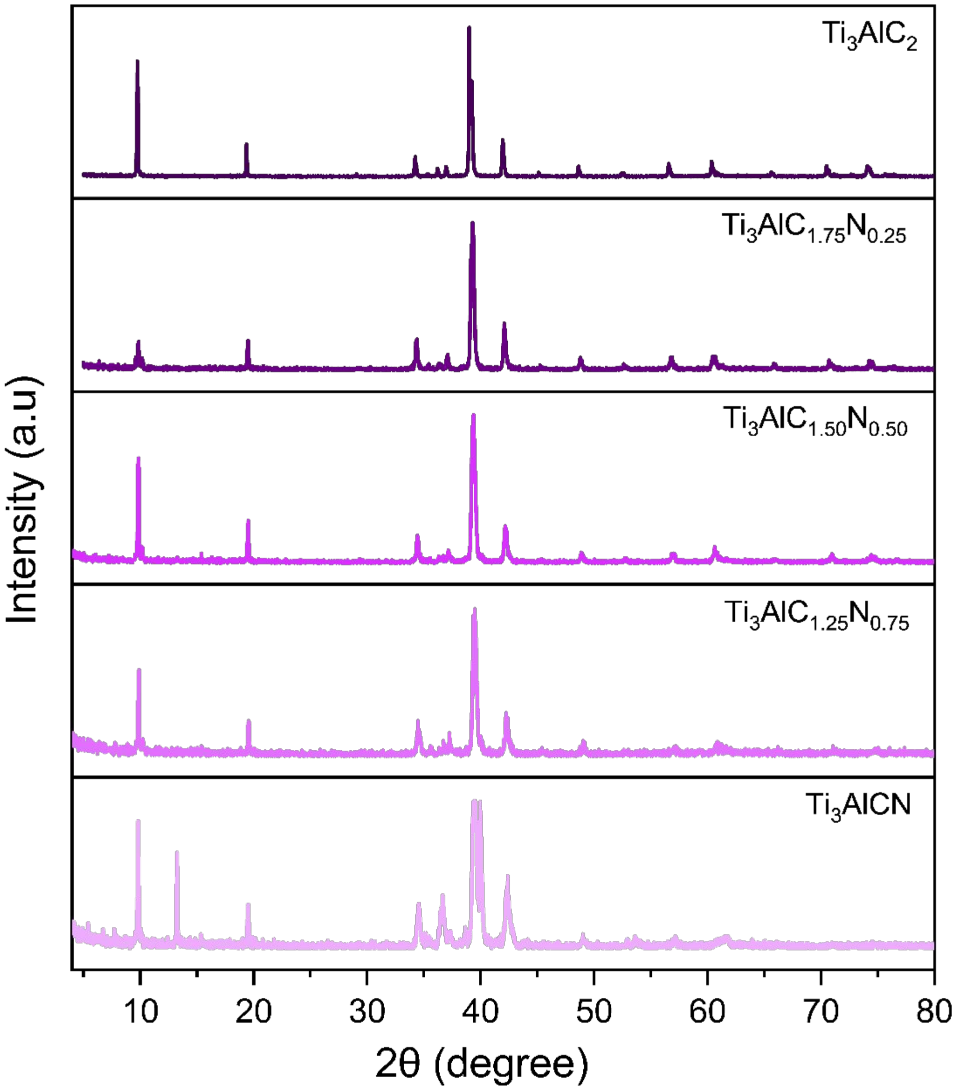

Figure S4. X-ray diffraction pattern of $Ti_3Al(C_{2-y}N_y)$ MAX phases for $y \leq 1$. The synthesis was done using stoichiometric powder ratios, ball-milling for 24 hours at 100 rpm, followed by annealing at 1450 °C for 4 hours under Ar.

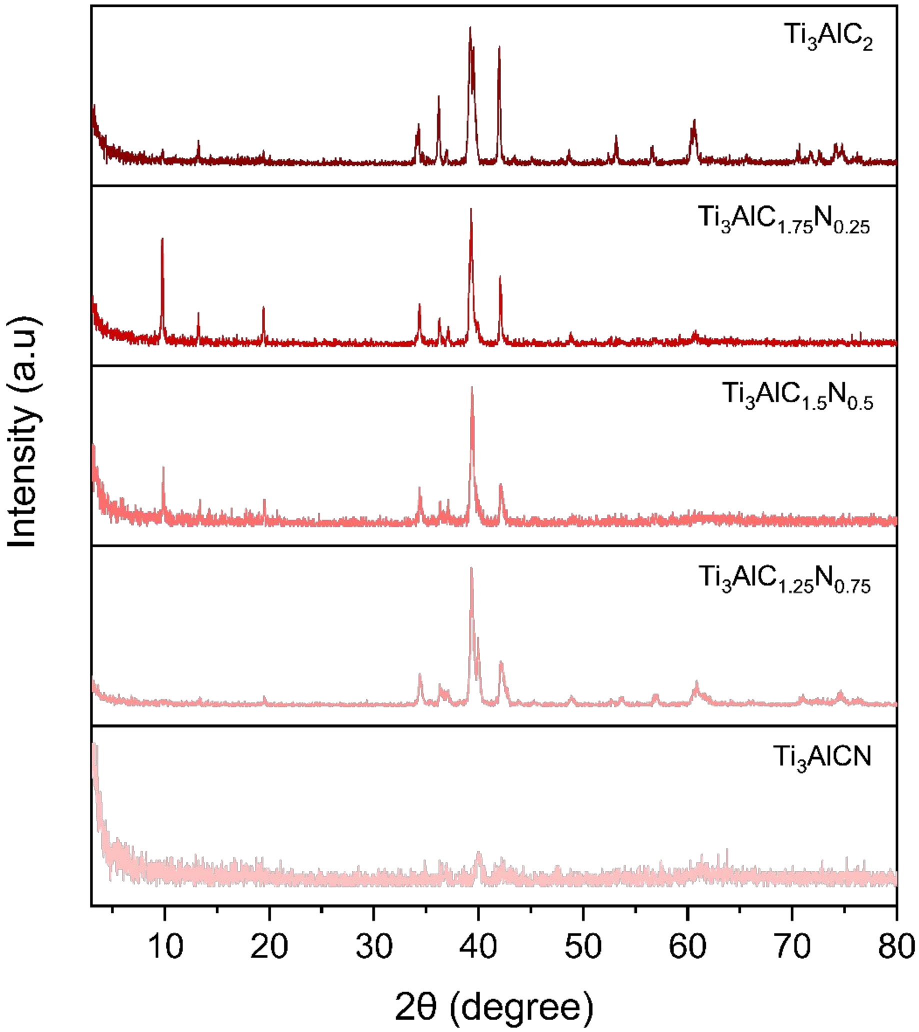

Figure S5. X-ray diffraction pattern of $Ti_3Al(C_{2-y}N_y)$ MAX phases for $y \leq 1$. The synthesis was done using stoichiometric powder ratios, ball-milling for 24 hours at 100 rpm, followed by annealing at 1400 °C for 4 hours under Ar.

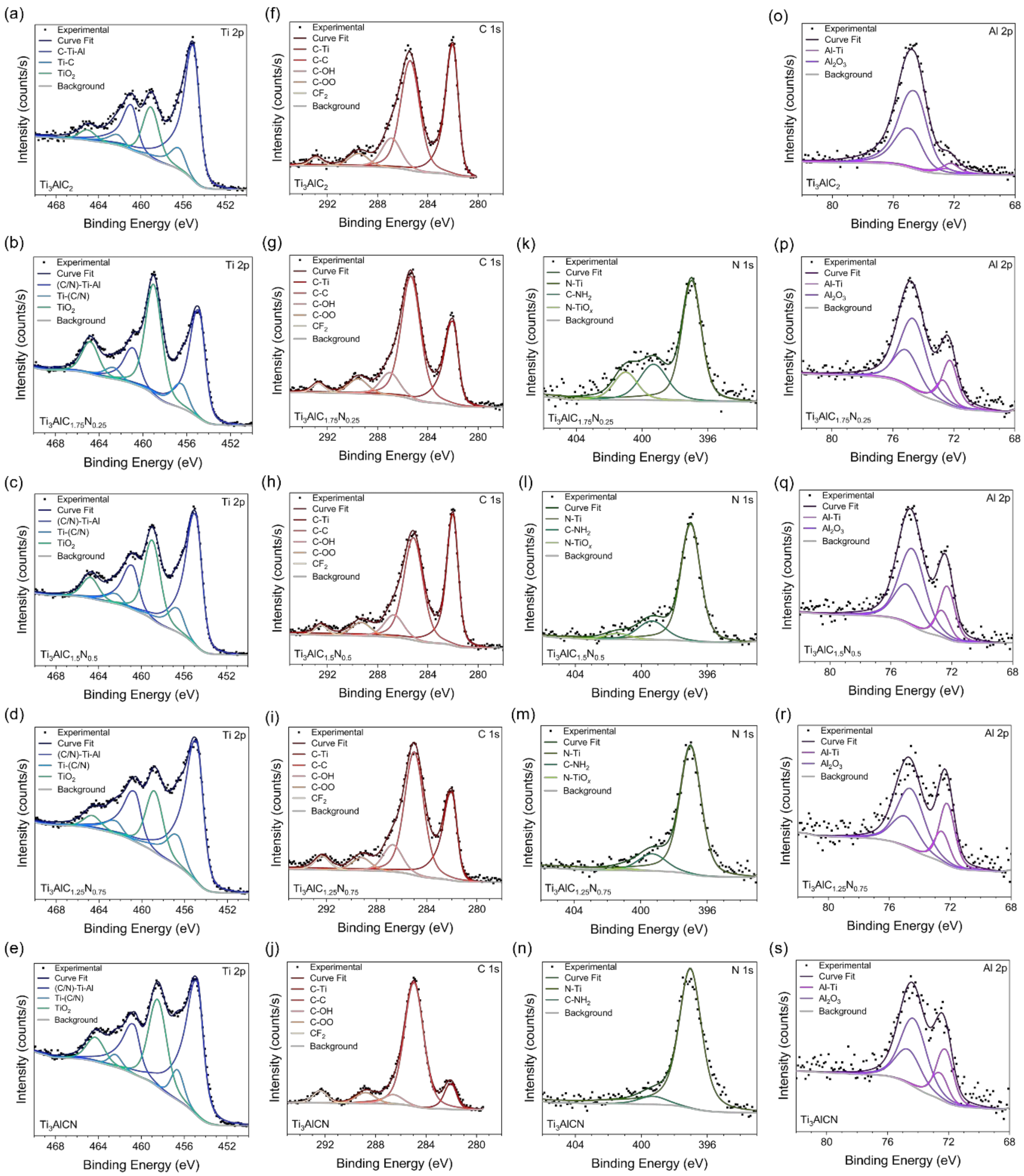

Figure S6. Fitted core-level XPS spectra of $Ti_3Al(C_{2-y}N_y)$ MAX phases. (a-e) Ti 2p, (f-j) C 1s, (k-n) N 1s, and (o-s) Al 2p regions.[3, 4]

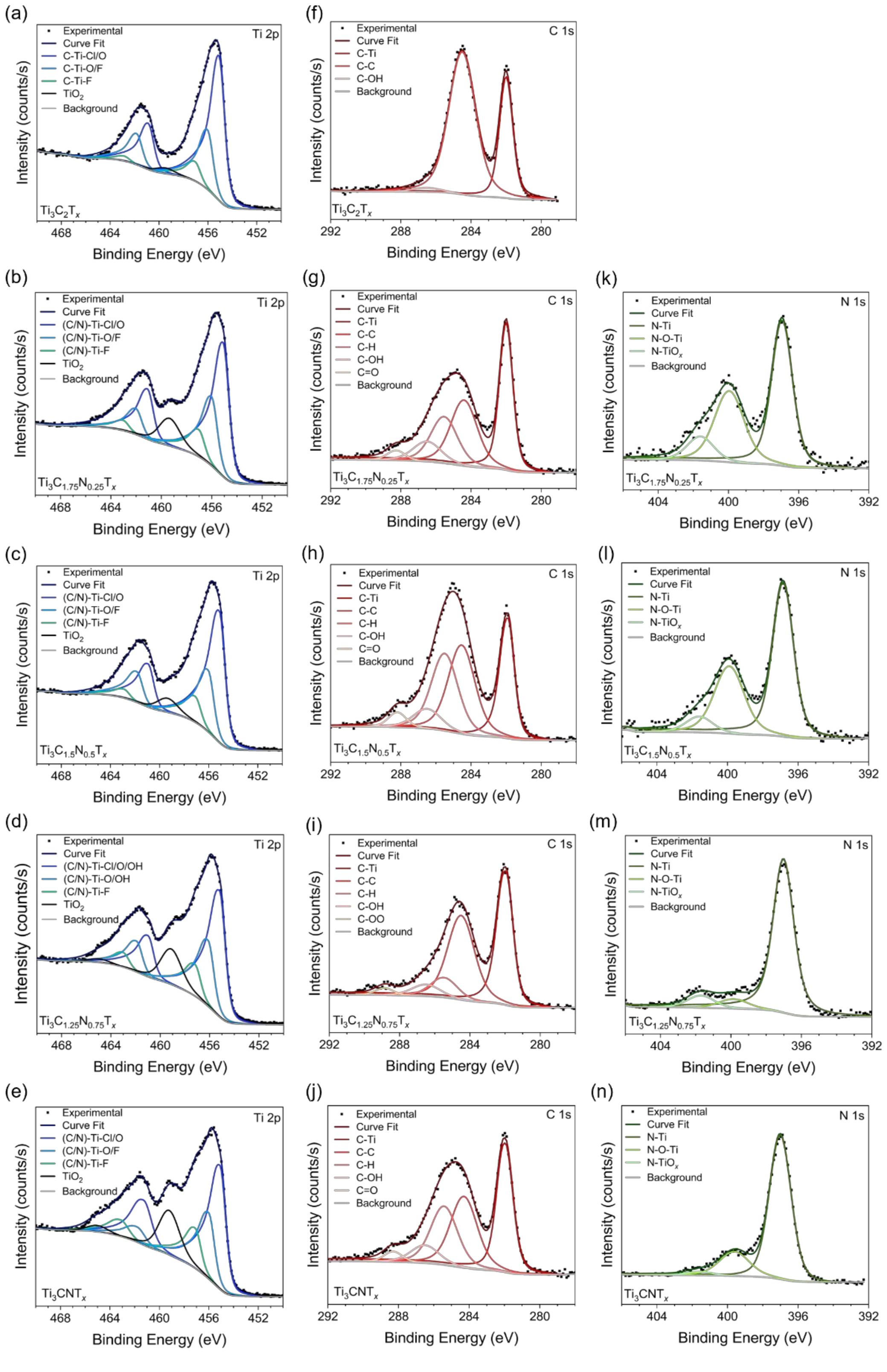

Figure S7. Fitted XPS core level spectra of $Ti_3(C_{2-y}N_y)T_x$ MXenes. (a-e) Ti 2p, (f-j) C 1s, and (k-n) N 1s regions.[5-7]

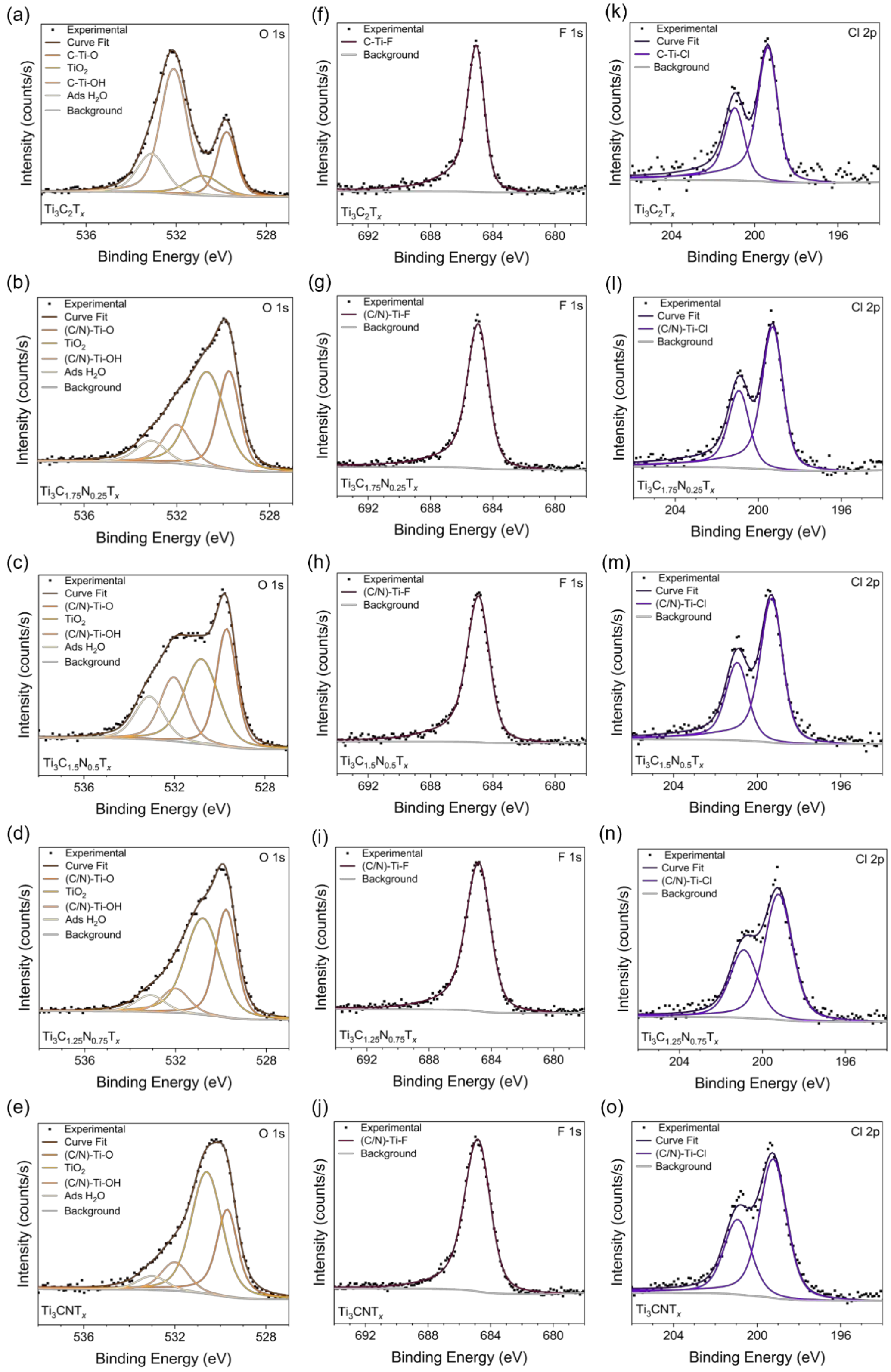

Figure S8. Fitted XPS core level spectra of $Ti_3(C_{2-y}N_y)T_x$ MXenes. (a-e) O 1s, (f-j) F 1s, and (k-o) Cl 2p regions.[3, 5]

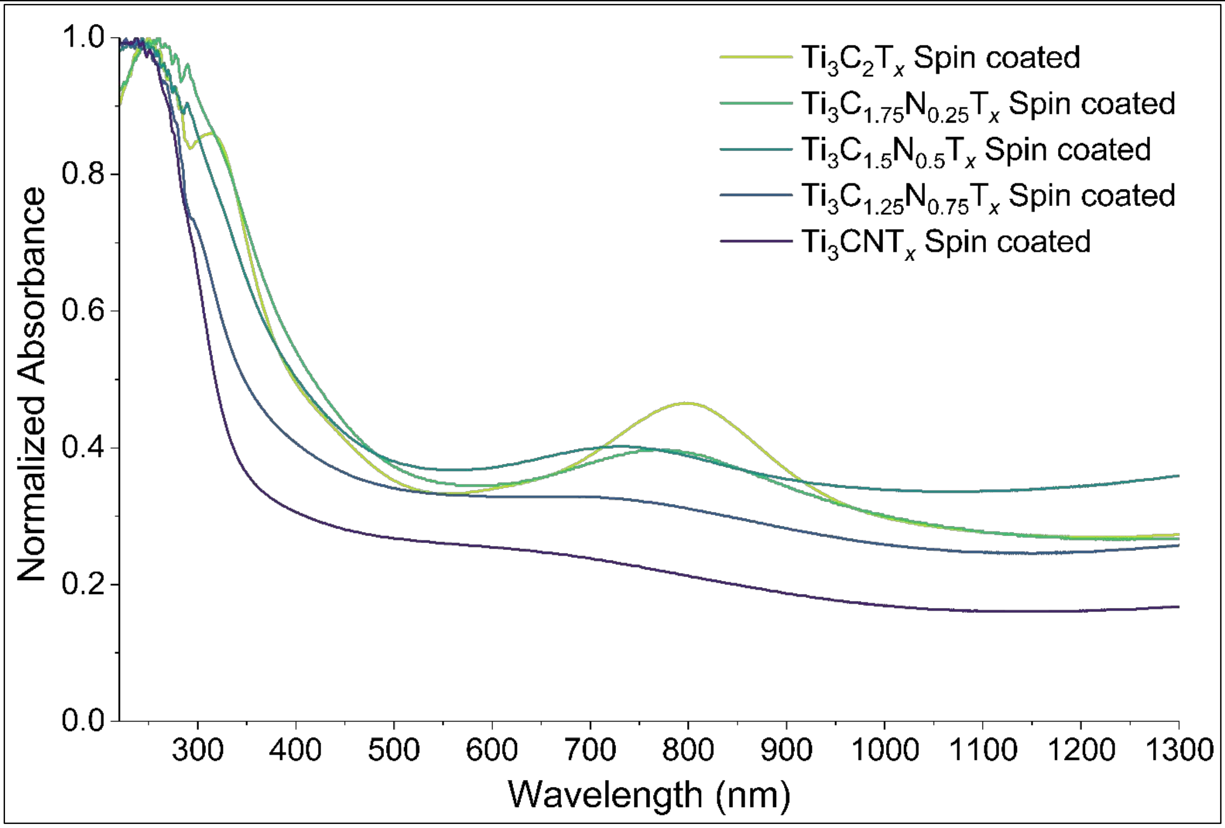

Figure S9. Optical absorption properties of $Ti_3(C_{2-y}N_y)T_x$ MXene thin films in 300-1300 nm. UV-Vis-NIR spectra were normalized to 1 using the maximum absorption of each MXene, which typically occurs at wavelengths of 200-300 nm.

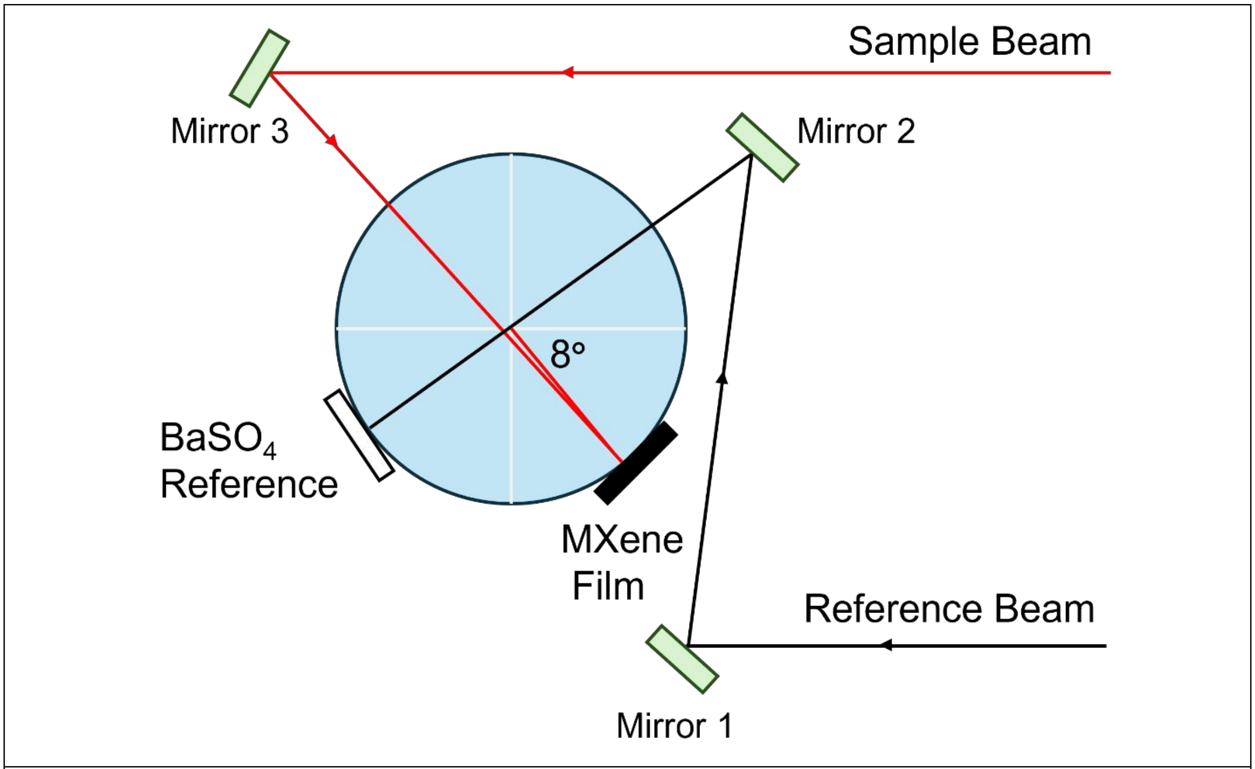

Figure S10. Schematic representation of the integrating sphere used for the total reflectance measurements. This geometry measures the sum of specular reflectance and diffuse reflectance. The total reflectance of MXene thin films spin-coated on a glass substrate was measured with respect to a reference ($BaSO_4$ pressed pellets).

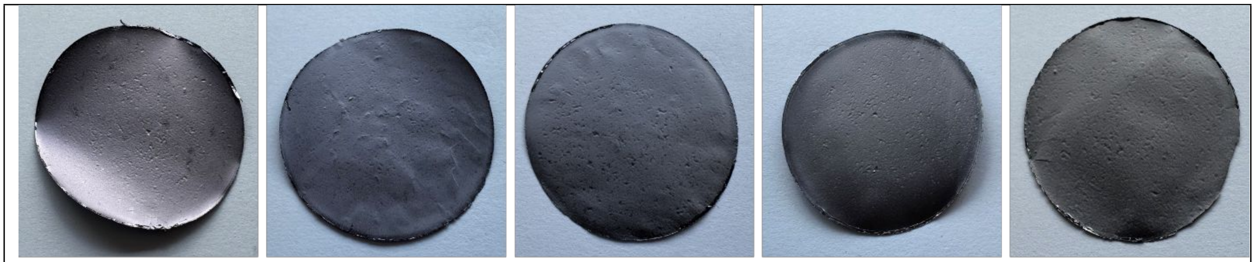

Figure S11. Free-standing $Ti_3(C_{2-y}N_y)T_x$ MXene films fabricated by vacuum filtration. A gradual shift in the reflected color from dark purple ($Ti_3C_2T_x$) to black (Ti_3CNT_x) was observed. The metallic luster of MXene films decreased from $Ti_3C_2T_x$ to Ti_3CNT_x, suggesting a decrease in free carrier concentrations.

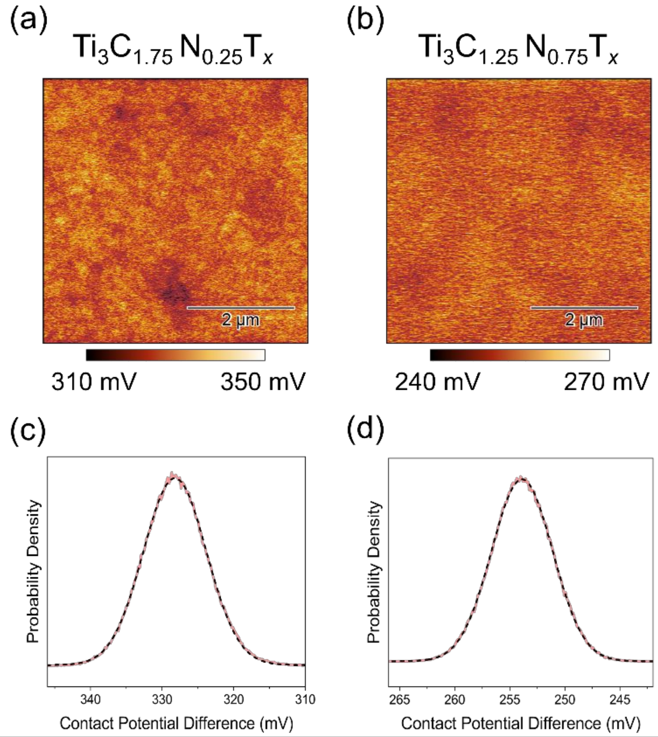

Figure S12. Spatially resolved surface potential distribution in $Ti_3(C_{2-y}N_y)T_x$ MXenes, measured using Kelvin probe force microscopy (a) $Ti_3C_{1.75}N_{0.25}T_x$, (b) $Ti_3C_{1.25}N_{0.75}T_x$. The scan area was 5×5 μm, and the scale bar represents the potential in millivolts (mV). Distribution of surface potentials compared to a Gaussian distribution (c) $Ti_3C_{1.75}N_{0.25}T_x$, (d) $Ti_3C_{1.25}N_{0.75}T_x$.

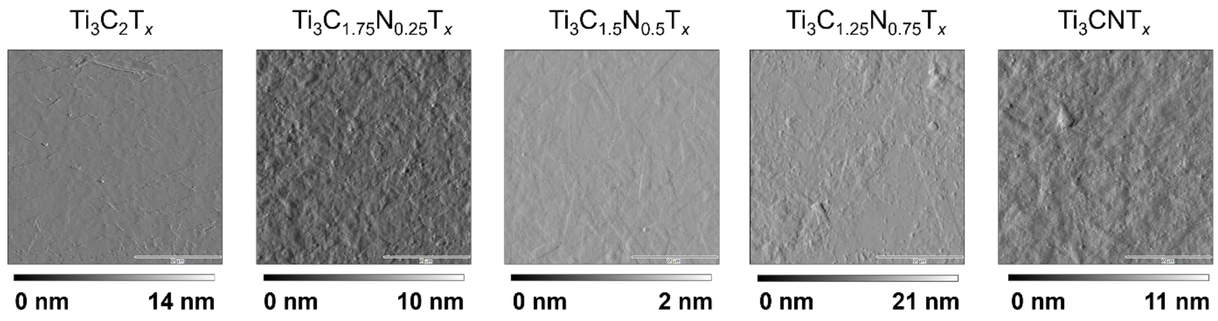

Figure S13. The topography of $Ti_3(C_{2-y}N_y)T_x$ MXenes spin-coated on glass slides was measured using the atomic force microscope in tapping mode.

Table S1. A summary of the Rietveld refinement results corresponding to the fits in Figure S2.

MAX Phase Composition	R_w	GOF	χ2	a-Lattice Parameter (Å)	c-Lattice Parameter (Å)
Ti_3AlC_2	42.812%	1.13	1.286	3.08422	18.62691
$Ti_3AlC_{1.75}N_{0.25}$	37.568%	1.21	1.455	3.06657	18.52489
$Ti_3AlC_{1.5}N_{0.5}$	35.399%	1.30	1.697	3.06054	18.46341
$Ti_3AlC_{1.25}N_{0.75}$	34.010%	1.35	1.817	3.05487	18.43263
Ti_3AlCN	33.388%	1.21	1.476	3.04356	18.40424

Table S2. C/N ratios in $Ti_3Al(C_{2-y}N_y)$ MAX phases measured in XPS.

MAX Phase	Nominal Carbon Composition	XPS Measured Carbon Composition	Nominal Nitrogen Composition	XPS Measured Nitrogen Composition
$Ti_3Al(C_{2-y}N_y)$	2	-	0	-
	1.75	1.69	0.25	0.31
	1.5	1.43	0.5	0.57
	1.25	1.18	0.75	0.82
	1	0.92	1	1.08

Table S3. C and N relative amounts in $Ti_3(C_{2-y}N_y)T_x$ MXene films measured in XPS.

MXene	Nominal Carbon Composition	XPS Measured Carbon Composition	Nominal Nitrogen Composition	XPS Measured Nitrogen Composition
$Ti_3(C_{2-y}N_y)T_x$	2	-	0	-
	1.75	1.69	0.25	0.31
	1.5	1.44	0.5	0.56
	1.25	1.26	0.75	0.74
	1	0.99	1	1.01

Table S4. Relative amounts (%) of F, Cl, and surface oxygen species (O+OH) obtained from XPS for the $Ti_3(C_{2-y}N_y)T_x$ MXene films, as a function of their nominal N content.

MXene	Nominal Nitrogen Composition	F	Cl	O/OH
$Ti_3(C_{2-y}N_y)T_x$	0	30.44	10.09	59.47
	0.25	47.57	16.88	35.55
	0.5	44.27	17.92	37.81
	0.75	44.42	17.76	37.82
	1	50.91	20.76	28.33

Table S5. XPS peak fitting and assignments for $Ti_3Al(C_{2-y}N_y)$ MAX phases.

Region	BE (eV)	Peak Assignment	References
Ti 2p $_{3/2}$ (2p $_{1/2}$)	454.9 (460.8)	C-Ti-Al	(3)
	456.6 (462.4)	C-Ti	(3)
	459.0 (464.8)	TiO_2	(3)
Al 2p $_{3/2}$ (2p $_{1/2}$)	72.3 (72.7)	Ti-Al	(3)
	74.6 (75.0)	Al_2O_3	(3)
C 1s	282.0	C-Ti	(4)
	285.2	C-C	(4)
	286.7	C-OH	(4)
	289.3	COO	(4)
	292.5	CF_2	(4)
N 1s	397.0	N-Ti	(4)
	399.4	$C-NH_2$	(4)
	401.2	N-TiO_x	(4)

Table S6. XPS peak fitting and assignments for $Ti_3(C_{2-y}N_y)T_x$ MXenes.

Region	BE (eV)	Peak Assignment	References
Ti 2p $_{3/2}$ (2p $_{1/2}$)	455.1 (461.0)	(C/N)-Ti-Cl/O	(5)
	456.0 (461.9)	(C/N)-Ti-O/F	(5)
	457.1 (463.1)	(C/N)-Ti-F	(5)
	459.2 (465.0)	TiO_2	(5)
C 1s	282.0	C-Ti	(5)
	284.5	C-C	(5)
	285.5	C-H	(4)
	286.5	C-OH	(7)
	288.3	C=O	(4)
	288.8	C-OO	(4)
N 1s	397.0	N-Ti	(6)
	399.9	N-O-Ti	(6)
	401.6	N-TiO_x	(6)
O 1s	529.8	C-Ti-O	(5)
	530.8	TiO_2	(5)
	532.0	C-Ti-OH	(5)
	533.1	Adsorbed H_2O	(5)
F 1s	684.9	C-Ti-F	(5)
Cl 2p $_{3/2}$ (2p $_{1/2}$)	199.3 (200.9)	C-Ti-Cl	(5)

**Table S7.** Summary of the electrical conductivity measurements of $Ti_3(C_{2-y}N_y)T_x$ carbonitride MXenes measured at a minimum of 10 different spots on the films.			
Composition	Mean Film Thickness (mm)	Mean Sheet Resistance (Ohms per square)	Mean Conductivity (S cm^{-1})
$Ti_3C_2T_x$	0.0112 ± 0.0001	0.0765 ± 0.0006	11710.7 ± 100.6
	0.0123 ± 0.0002	0.0798 ± 0.0004	10213.6 ± 191.1
	0.0139 ± 0.0007	0.0770 ± 0.0008	9332.7 ± 532.8
	0.0137 ± 0.0002	0.0775 ± 0.0005	9388.5 ± 160.9
$Ti_3C_{1.75}N_{0.25}T_x$	0.0059 ± 0.0002	0.2551 ± 0.0018	6613.2 ± 208.8
	0.0154 ± 0.0002	0.1064 ± 0.0005	6117.6 ± 103.3
	0.0126 ± 0.0002	0.1579 ± 0.0008	5033.6 ± 89.9
$Ti_3C_{1.5}N_{0.5}T_x$	0.0086 ± 0.0002	0.2902 ± 0.0017	3994.4 ± 85.6
	0.0099 ± 0.0001	0.2547 ± 0.0010	3965.8 ± 20.3
	0.0105 ± 0.0001	0.3160 ± 0.0029	3016.9 ± 32.6
$Ti_3C_{1.25}N_{0.75}T_x$	0.0061 ± 0.0001	0.7145 ± 0.0043	2287.6 ± 50.8
	0.0056 ± 0.0001	0.8091 ± 0.0038	2199.9 ± 30.3
	0.0085 ± 0.0001	0.7666 ± 0.0029	1536.2 ± 26.2
Ti_3CNT_x	0.0110 ± 0.0001	0.7269 ± 0.0035	1253.7 ± 8.9
	0.0126 ± 0.0001	0.7089 ± 0.0042	1117.1 ± 9.8
	0.0083 ± 0.0001	1.1032 ± 0.0143	1086.2 ± 17.8
	0.0061 ± 0.0001	1.6627 ± 0.0195	991.8 ± 14.8

$$\text{Bulk resistivity } (\rho) = \text{Sheet resistance } (R_s) \times \text{Thickness} \qquad \textbf{equation 1}$$

$$\text{Bulk conductivity } (\sigma) = \frac{1}{\text{Bulk resistivity } (\rho)} \qquad \textbf{equation 2}$$

References

(1) Toby, B. H.; Von Dreele, R. B., Gsas-Ii: The Genesis of a Modern Open-Source All Purpose Crystallography Software Package. *Journal of Applied Crystallography* **2013,** 46, (2), 544-549.
(2) Shekhirev, M.; Shuck, C. E.; Sarycheva, A.; Gogotsi, Y., Characterization of Mxenes at Every Step, from Their Precursors to Single Flakes and Assembled Films. *Progress in Materials Science* **2021,** 120, 100757.
(3) Zhang, T.; Shuck, C. E.; Shevchuk, K.; Anayee, M.; Gogotsi, Y., Synthesis of Three Families of Titanium Carbonitride MXenes. *Journal of the American Chemical Society* **2023,** 145, (41), 22374-22383.
(4) Hantanasirisakul, K.; Alhabeb, M.; Lipatov, A.; Maleski, K.; Anasori, B.; Salles, P.; Ieosakulrat, C.; Pakawatpanurut, P.; Sinitskii, A.; May, S. J.; Gogotsi, Y., Effects of Synthesis and Processing on Optoelectronic Properties of Titanium Carbonitride MXene. *Chemistry of Materials* **2019,** 31, (8), 2941-2951.
(5) Natu, V.; Benchakar, M.; Canaff, C.; Habrioux, A.; Célérier, S.; Barsoum, M. W., A Critical Analysis of the X-Ray Photoelectron Spectra of $Ti_3C_2T_z$ MXenes. *Matter* **2021,** 4, (4), 1224-1251.
(6) Jindata, W.; Hantanasirisakul, K.; Eknapakul, T.; Denlinger, J. D.; Sangphet, S.; Chaiyachad, S.; Jaisuk, C.; Rasritat, A.; Sawasdee, T.; Nakajima, H.; Rattanachata, A.; Fongkaew, I.; Limpijumnong, S.; Gogotsi, Y.; Meevasana, W., Spectroscopic Signature of Negative Electronic Compressibility from the Ti Core-Level of Titanium Carbonitride MXene. *Applied Physics Reviews* **2021,** 8, (2), 021401.
(7) Näslund, L.-Å.; Persson, P. O. Å.; Rosen, J., X-Ray Photoelectron Spectroscopy of Ti_3AlC_2, $Ti_3C_2T_z$, and TiC Provides Evidence for the Electrostatic Interaction between Laminated Layers in MAX-Phase Materials. *The Journal of Physical Chemistry C* **2020,** 124, (50), 27732-27742.
